# ﻿Two novel species of *Neomonodictys* and *Phaeoisaria* (Pleurotheciaceae, Pleurotheciales) from freshwater habitats in China

**DOI:** 10.3897/mycokeys.122.164339

**Published:** 2025-09-16

**Authors:** Wang-ming Zhang, Qin-ying Feng, Xiao-yu Song, Wan-qing Xie, Xin-zhong Zhou, Juan Lu, Li Lu

**Affiliations:** 1 Beijing Jishuitan Hospital Guizhou Hospital, Guiyang 550014, China Beijing Jishuitan Hospital Guizhou Hospital Guiyang China

**Keywords:** Asexual morph, phylogeny, Sordariomycetes, taxonomy

## Abstract

During a recent survey of lignicolous freshwater fungi in Guizhou Province, China, two fungi were collected from submerged woody substrates and were preliminarily identified as members of the family Pleurotheciaceae. Comprehensive morphological examinations, combined with multi-locus phylogenetic analyses based on ITS, LSU, SSU, and *rpb2* sequence data, revealed that both specimens represent previously undescribed taxa. In this study, we provide detailed morphological descriptions, illustrations, and phylogenetic evidence to support the establishment of two novel species, *Neomonodictys subtropicus* and *Phaeoisaria guiyangensis*. The discovery of these taxa contributes to the growing body of knowledge on the diversity and taxonomy of freshwater fungi in Southwest China. It also emphasizes the ecological importance of Pleurotheciaceae in lignicolous freshwater habitats and highlights the potential for discovering novel fungal lineages in underexplored regions such as Guizhou Province.

## ﻿Introduction

Lignicolous freshwater fungi are those that colonize woody substrates in a variety of freshwater habitats (Luo et al. 2004; Hyde et al. 2016; Calabon et al. 2022, 2023). These species play a crucial role in the decomposition of lignocellulosic material, the release of nutrients, and the maintenance of biodiversity in freshwater ecosystems (Yuen et al. 1998; Bucher et al. 2004; Hyde et al. 2016). Asia is among the most active regions for freshwater fungal research, particularly in China (Guizhou, Hainan, Hong Kong, Taiwan, and Yunnan) and northern Thailand (Chiang Mai and Chiang Rai), where a large number of species have been documented (Zhang et al. 2011; Jones et al. 2014; Hyde et al. 2016, 2021; Su et al. 2016; Bao et al. 2018, 2019, 2020, 2021, 2023; Luo et al. 2018b, 2019; Dong et al. 2020, 2021a, b; Calabon et al. 2021, 2022, 2023; Shen et al. 2022a, b, 2024; Fryar and Catcheside 2023; Fryar et al. 2023; Xu et al. 2023, 2024b, 2025; Yang et al. 2023; Ma et al. 2024b; Wang et al. 2024a, b; Xiao et al. 2025).

Pleurotheciaceae was established by Réblová et al. (2016), with *Pleurothecium* as the type genus. Both sexual and asexual morphs of Pleurotheciaceae have been reported, with the sexual morphs characterized by dark, papillate, perithecial, astromatic, immersed to superficial ascomata; unitunicate asci with a distinct non-amyloid apical annulus; and fusiform to ellipsoidal, septate, hyaline ascospores (Réblová et al. 2016; Luo et al. 2018a; Hyde et al. 2020). The asexual morphs of Pleurotheciaceae include species with diverse morphologies, comprising acrodictys-like (Hyde and Hyde 2002; Sadowski et al. 2012), helicoön-like (Dayarathne et al. 2019; Réblová et al. 2020), monodictys-like (Hyde et al. 2020), and dactylaria-like taxa (Réblová et al. 2016; Luo et al. 2018a). Currently, the family Pleurotheciaceae comprises 16 genera, including *Adelosphaeria*, *Anapleurothecium*, *Coleodictyospora*, *Dematipyriforma*, *Helicoascotaiwania*, *Melanotrigonum*, *Monotosporella*, *Neomonodictys*, *Obliquifusoideum*, *Phaeoisaria*, *Phragmocephala*, *Pleurotheciella*, *Pleurothecium*, *Pseudosaprodesmium*, *Rhexoacrodictys*, and *Sterigmatobotrys* (Hyde et al. 2024; Wang et al. 2024a, 2025). To date, more than 100 species have been accepted within Pleurotheciaceae, most of which occur as saprobes on various plant substrates in both terrestrial and freshwater habitats (Réblová et al. 2016; Luo et al. 2018a; Dong et al. 2021b; Shi et al. 2021; Bao et al. 2022; Huang et al. 2022; Wang et al. 2024a; Xu et al. 2024a, 2025).

*Phaeoisaria* was established by Höhnel (1909), with *P. bambusae* as the type species. *Phaeoisaria* species are primarily known from their asexual morphs, which are characterized by erect synnemata with parallel adpressed conidiophores; polyblastic, sympodially conidiogenous cells with denticulation; and ellipsoidal or obovoidal, aseptate or septate conidia (de Hoog and Papendorf 1976; Réblová et al. 2016; Luo et al. 2018a). The first sexual morph was characterized by immersed, globose-to-elongate ascomata with a long, cylindrical, black ostiolar neck; filamentous, branched, septate paraphyses; unitunicate, cylindrical asci with a small refractive apical apparatus; and hyaline, filiform, multi-septate ascospores with tapering ends (Luo et al. 2018a; Wang et al. 2024a). Members of *Phaeoisaria* occur primarily as saprobes in plant debris, soils, and sediments in terrestrial and freshwater habitats (Cheng et al. 2014; Réblová et al. 2016; Crous et al. 2017, 2021; Luo et al. 2018a; Hyde et al. 2019; Wang et al. 2024a).

*Neomonodictys* was established by Hyde et al. (2020) to accommodate the asexual species *N. muriformis*, which was collected from decaying wood in a freshwater habitat in Thailand. Subsequently, Huang et al. (2022) described the second species, *N. aquatica*, from submerged wood in a lake on the Yunnan Plateau in China. To date, only asexual morphs have been reported for this genus, which is characterized by the absence of conidiophores; holoblastic, monoblastic, integrated, terminal, determinate conidiogenous cells; and acrogenous, solitary, subglobose to globose, muriform conidia (Hyde et al. 2020; Huang et al. 2022). Both known species are saprobic and occur on decaying wood in freshwater habitats, with records currently limited to China and Thailand (Hyde et al. 2020; Huang et al. 2022).

During our investigation of freshwater, wood-inhabiting fungi in Guizhou Province, China, we collected two specimens belonging to the family Pleurotheciaceae. Detailed morphological examinations and multi-gene phylogenetic analyses were conducted, leading to the identification and description of two novel species, which are formally described and illustrated in this study.

## ﻿Materials and methods

### ﻿Sample collection and specimen examination

Fresh specimens were collected from freshwater habitats in Guiyang City and Zunyi City, Guizhou Province, China. Samples were taken to the laboratory in plastic bags, labeled with collection details, including locality, habitat, and date (Rathnayaka et al. 2024). Specimens from freshwater habitats were cultured at room temperature and maintained in a moist environment for about two weeks. The samples were examined using a stereomicroscope (SMZ 745, Nikon, Japan). Micro-morphological characters were captured using a Nikon EOS 90D digital camera attached to an ECLIPSE Ni compound microscope (Nikon, Japan). Measurements of conidiophores, conidiogenous cells, and conidia were carried out using the Tarosoft (R) Image Frame Work program.

### ﻿Isolation and material deposition

Single-spore isolation was performed following the method described by Senanayake et al. (2020). The germinated conidia were aseptically transferred to fresh potato dextrose agar (PDA) and incubated at room temperature for 29–35 days. Morphological characteristics of the fungal mycelium on PDA, including color, shape, size, margin, and elevation, were documented.

Dried fungal specimens were deposited in the ` Herbarium of Guizhou Academy of Agriculture Sciences (Herb. GZAAS), Guiyang, China. Pure cultures were deposited in the Guizhou Culture Collection (GZCC), Guiyang, China. Descriptions of the new taxa were uploaded to the Faces of Fungi webpage following the guidelines of Jayasiri et al. (2015). The new species were registered in the MycoBank database (https://www.mycobank.org/), and MycoBank numbers were obtained.

### ﻿DNA extraction, PCR amplification, and sequencing

Fresh fungal mycelia grown on PDA were scraped using sterilized scalpels. Genomic DNA was extracted using the Biospin Fungus Genomic DNA Extraction Kit (BioFlux, China), following the manufacturer’s protocol. The primer pairs ITS5/ITS4 (White et al. 1990), LR0R/LR5 (Vilgalys and Hester 1990), NS1/NS4 (White et al. 1990), and fRPB2-5F/fRPB2-7cR (Liu et al. 1999) were used to amplify the ITS, LSU, SSU, and *rpb2* regions, respectively. PCR amplification was performed in a 25 μL reaction volume, consisting of 13.5 μL of 10× PCR Master Mix, 1 μL of each primer, 1 μL of template DNA, and 8.5 μL of ddH_2_O. The thermocycling conditions were as follows: initial denaturation at 94 °C for 3 min; 40 cycles of denaturation at 94 °C for 45 s, annealing at 56 °C for 50 s, and extension at 72 °C for 1 min; followed by a final extension at 72 °C for 10 min. The PCR products were purified and sequenced by Sangon Biotech (Shanghai, China) Co., Ltd.

### ﻿Phylogenetic analyses

BioEdit v. 7.0.5.3 (Hall 1999) and SeqMan v. 7.0.0 (Swindell and Plasterer 1997) were used to check and assemble the newly generated sequences. The sequences incorporated in this study were downloaded from GenBank (Table 1; https://www.ncbi.nlm.nih.gov/). Multiple sequence alignments for each locus dataset were performed using MAFFT v. 7.473 (https://mafft.cbrc.jp/alignment/server/, Katoh et al. 2019) and visually inspected in AliView (Larsson 2014). The ITS, LSU, SSU, and *rpb2* alignments were trimmed using trimAl v. 1.2rev59 (Capella-Gutiérrez et al. 2009) and subsequently merged using SequenceMatrix v. 1.7.8 (Vaidya et al. 2011).

**Table 1.** Taxa used in the phylogenetic analyses, along with their corresponding GenBank accession numbers.

Taxa	Strain numbers	GenBank accession numbers			
		ITS	LSU	SSU	*rpb*2
*Adelosphaeria catenata*	CBS 138679^T^	KT278707	KT278707	KT278692	KT278743
*Anapleurothecium botulisporum*	FMR 11490^T^	KY853423	KY853483	–	–
*Ascotaiwania fusiformis*	MFLU 15-1156^T^	MG388215	NG_057114	–	–
*Canalisporium grenadoideum*	SS 03615^T^	–	GQ390267	GQ390252	HQ446420
*Coleodictyospora muriformis*	MFLUCC 18-1243	MW981642	MW981648	MW981704	–
*Conioscypha lignicola*	CBS 335.93^T^	–	AY484513	JQ437439	JQ429260
*C. minutispora*	CBS 137253^T^	–	MH878131	–	–
*Dematipyriforma aquilaria*	CGMCC 3.17268^T^	KJ138621	KJ138623	KJ138622	–
*D. muriformis*	MFLU 21-0146^T^	OM654773	OM654770	–	–
*Helicoascotaiwania lacustris*	CBS 145963^T^	MN699399	MN699430	MN699382	MN704304
*Melanotrigonum ovale*	CBS 138742^T^	KT278723	KT278708	KT278695	KT278744
*Monotosporella setosa*	HKUCC 3713	–	AF132334	–	–
*Neomonodictys aquatica*	KUNCC 21-10708^T^	MZ686200	OK245417	–	–
*N. muriformis*	MFLUCC 16-1136	MN644509	MN644485	–	–
***N. subtropicus***	**GZCC 25-0628^T^**	**PV871231**	**PV871237**	**PV871239**	**PV872882**
***N. subtropicus***	**GZCC 25-0629**	**PV871232**	**PV871238**	**PV871240**	**PV872883**
*Obliquifusoideum triseptatum*	CGMCC 3.27014^T^	PP445243	PP049503	PP049521	PP068779
*Phaeoisaria loranthacearum*	BYCDW25	MG820097	–	–	–
*P. loranthacearum*	BYCDW24	MG820098	–	–	–
*P. pseudoclematidis*	MFLUCC 11-0393^T^	KP744457	KP744501	KP753962	–
*P. sedimenticola*	CGMCC 3.14949^T^	JQ074237	JQ031561	–	–
*P. annesophieae*	CBS 143235^T^	MG022180	MG022159	–	–
*P. annesophieae*	MFLU 19-0531	MT559109	MT559084	–	–
*P. aquatica*	MFLUCC 16-1298^T^	MF399237	MF399254	–	MF401406
*P. clematidis*	MFLUCC 16-1273	MF399229	MF399246	–	–
*P. clematidis*	MFLUCC 17-1341	MF399230	MF399247	MF399216	MF401400
*P. clematidis*	MFLUCC 17-1968	MG837022	MG837017	MG837027	–
*P. clematidis*	DAOM 226789	JQ429155	JQ429231	JQ429243	JQ429262
*P. dalbergiae*	CPC 39540^T^	OK664703	OK663742	OK663796	OK651159
*P. ellipsoidea*	IFRDCC 31^3^4T	ON533383	ON533387	–	–
*P. fasciculata*	CBS 127885^T^	KT278719	KT278705	KT278693	KT278741
*P. fasciculata*	DAOM 230055	KT278720	KT278706	KT278694	KT278742
*P. filiformis*	MFLUCC 18-0214^T^	MK878381	MK835852	MK834785	–
*P. goiasensis*	FCCUFG 02^T^	MT210320	MT375865	–	–
*P. goiasensis*	FCCUFG 03	MT210321	MT375866	–	–
*P. guttulata*	MFLUCC 17-1965^T^	MG837021	MG837016	MG837026	–
***P. guiyangensis***	**GZCC 25-0626^T^**	**PV871233**	–	**PV871241**	**PV872884**
***P. guiyangensis***	**GZCC 25-0627**	**PV871234**	–	**PV871242**	**PV872885**
*P. laianensis*	JAUCC4967^T^	ON937559	ON937557	ON937562	–
*P. laianensis*	JAUCC4968	ON937560	ON937561	ON937558	–
*P. loranthacearum*	CBS 140009^T^	KR611888	MH878676	–	–
*P. microspora*	MFLUCC 16-0033^T^	MF671987	MF167351	–	MF167352
*P. motuoensis*	KUNCC 10410^T^	OP626333	OQ947034	OQ947036	–
*P. obovata*	CGMCC 3.27015^T^	PP049488	PP049504	PP049522	PP068788
*P. sedimenticola*	S-908	MK878380	MK835851	–	–
*P. siamensis*	MFLUCC 16-0607^T^	MK607610	MK607613	MK607612	MK607611
*P. sparsa*	FMR 11939	–	HF677185	–	–
*P.* sp.	GZCC 22-2055	OR427331			
*P. synnematica*	NFCCI 4479^T^	MK391494	MK391492	–	–
*Phragmocephala stemphylioides*	DAOM 673211	KT278730	KT278717	–	–
*Pleurotheciella saprophytica*	MFLUCC 16-1251^T^	MF399241	MF399258	MF399224	MF401410
*P. submersa*	MFLUCC 17-1709^T^	MF399243	MF399260	MF399226	MF401412
*Pleurothecium pulneyense*	MFLUCC 16-1293^T^	–	MF399262	MF399228	MF401414
*P. semifecundum*	CBS 131271	JQ429159	JQ429240	JQ429254	JQ429270
*Pseudosaprodesmium cocois*	MFLU 23-0225^T^	OR438401	OR438864	OR458363	–
*Rhexoacrodictys melanospora*	KUNCC 22-12406^T^	OP168085	OP168087	OP168088	OP208807
*Saprodesmium dematiosporium*	KUMCC 18-0059^T^	MW981646	MW981647	MW981707	–
*Sterigmatobotrys macrocarpa*	DAOM 230059	JQ429154	GU017316	–	–
*S. macrocarpa*	PRM 915682	JQ429153	GU017317	JQ429255	–
*S. rudis*	DAOM 229838	JQ429152	JQ429241	JQ429256	JQ429272

Note: “^T^” indicates ex-type strains. Newly generated sequences are in bold black. “-” indicates the unavailable data in GenBank.

Maximum likelihood (ML) analysis was conducted using the IQ-TREE web server (http://iqtree.cibiv.univie.ac.at/) based on Bayesian Information Criteria (BIC) (Nguyen et al. 2015). The substitution model was automatically selected by the server. Bayesian inference (BI) analysis was performed using MrBayes on XSEDE (3.2.7a) via CIPRES Science Gateway (Stamatakis 2014). The aligned FASTA file was converted to NEXUS format using AliView (Larsson 2014). The best-fit evolutionary model for each dataset was determined using MrModeltest v. 2.3.10 (Nylander 2004). The posterior probabilities (BYPP) were determined based on Bayesian Markov chain Monte Carlo (BMCMC) sampling (Huelsenbeck and Ronquist 2001). Four simultaneous Markov chains were run for 10,000,000 generations, and trees were sampled every 1,000^th^ generation. The burn-in phase was set at 25%, and the remaining trees were used to calculate posterior probabilities.

Phylogenetic trees were visualized using FigTree v. 1.4.4 and further edited in PowerPoint. The photo plates were made using Adobe Photoshop CS6 software (Adobe Systems, USA).

CBS: CBS Fungal Biodiversity Centre, Centraalbureau voor Schimmelcultures, Utrecht, The Netherlands; CPC: Collection Pedro Crous, housed at CBS; CGMCC: China General Microbiological Culture Collection Center, Beijing, China; DAOM: Canadian National Mycological Herbarium; FMR: Facultad de Medicina, Reus, Tarragona, Spain; FCCUFG: Coleção de Culturas de Fungos da Universidade Federal de Goiás, Brazil; GZCC: Guizhou Culture Collection, Guiyang, China; HKUCC: University of Hong Kong Culture Collection, Department of Ecology and Biodiversity, Hong Kong, China; IFRDCC: International Fungal Research & Development Centre Culture Collection; JAUCC: Jiangxi Agricultural University Culture Collection; KUMCC: Kunming Institute of Botany Culture Collection; KUNCC: Cryptogams Kunming Institute of Botany, Academia Sinica, China; MFLUCC: Culture Collection of Mae Fah Luang University (MFLU), Chiang Rai, Thailand; NFCCI: National Fungal Culture Collection of India; PRM: Mycological Herbarium, National Museum, Prague, Czech Republic.

### ﻿Phylogenetic analysis results

**Figure 1.**
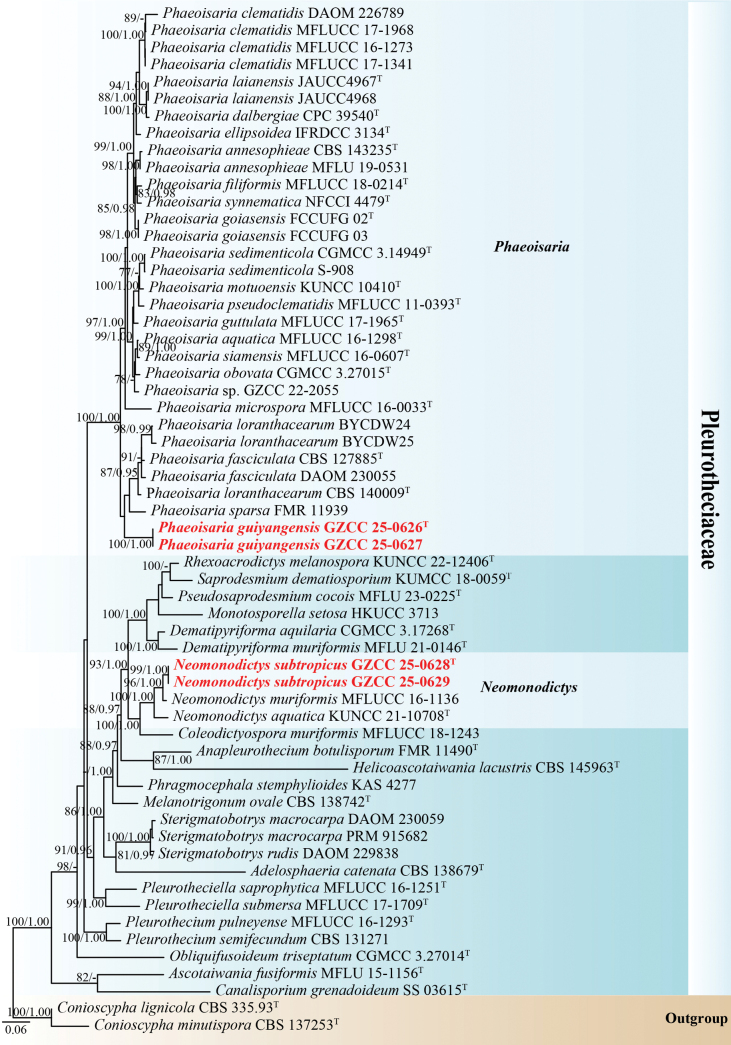
Phylogenetic tree generated from ML analysis based on the combined ITS, LSU, SSU, and *rpb2* sequence data. Bootstrap support values for ML (≥ 75%) and BI (≥ 0.95) are indicated near their respective nodes. The tree is rooted with *Conioscypha lignicola* (CBS 335.93) and *C. minutispora* (CBS 137253). Ex-type strains are denoted with “^T,^” and newly obtained strains are in bold red fonts.

The phylogenetic placements of the four new strains were determined by multi-locus phylogenetic analysis. The concatenated sequence matrix comprised 3,495 characters (ITS: 1–611, LSU: 612–1,471, SSU: 1,472–2,455, and *rpb2*: 2,456–3,495) across 60 taxa. Both ML and BI analyses produced congruent topologies. Fig. 1 presents the best-scoring ML tree, which had a final log-likelihood value of –26,978.362.

Based on the multi-locus phylogenetic analyses (Fig. 1), our collections belong to *Neomonodictys* and *Phaeoisaria* within Pleurotheciaceae (Pleurotheciales, Sordariomycetes). Two isolates (GZCC 25-0628 and GZCC 25-0629) formed a sister clade with *Neomonodictys muriformis* (MFLUCC 16-1136), supported by 96% ML and 1.00 BYPP. Additionally, our new isolates (GZCC 25-0626 and GZCC 25-0627) clustered together with a clade comprising *Phaeoisaria fasciculata* (CBS 127885 and DAOM 230055), *P. loranthacearum* (BYCDW24, BYCDW25, and CBS 140009), and *P. sparsa* (FMR 11939).

## ﻿Taxonomy

### ﻿ Neomonodictys subtropicus

Taxon classificationFungiPleurothecialesPleurotheciaceae

W.M. Zhang & L. Lu sp. nov.

3EEF9C5F-AF99-5C9C-826B-D1B1ECE05378

904153

Fig. 2

#### Etymology.

The specific epithet “*subtropicus*” refers to the subtropical climate of the collection site.

**Figure 2.**
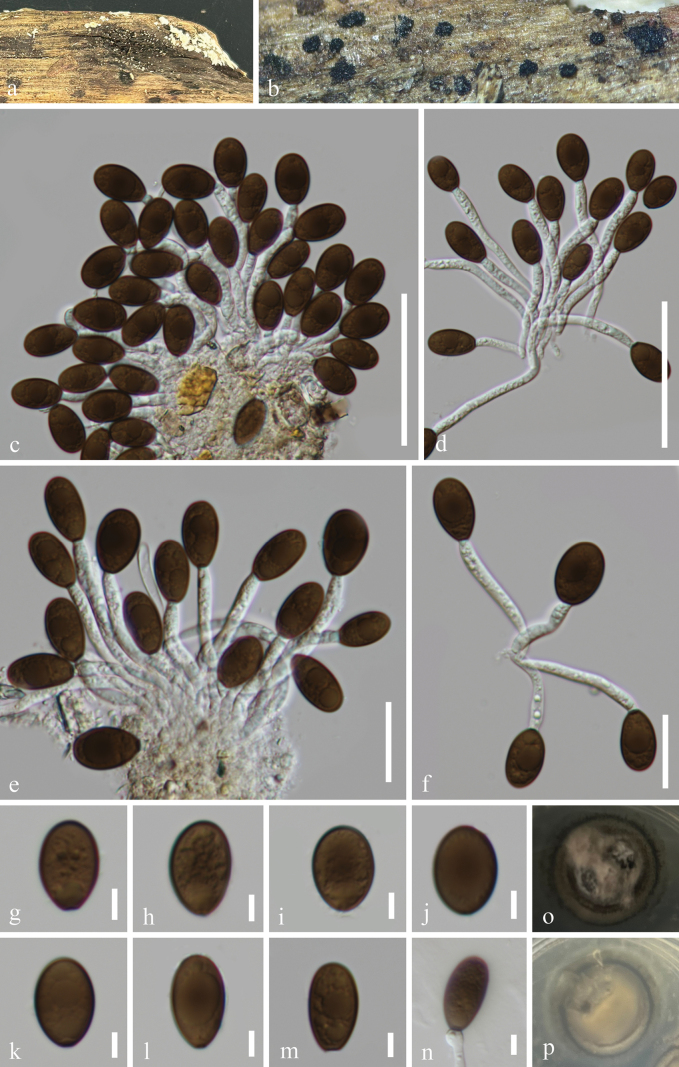
*Neomonodictys subtropicus* (GZAAS 25-0656, holotype). a, b. Colonies on the host surface; c–f. Conidiophores, conidiogenous cells, and conidia; g–m. Conidia; n. Germinated conidium; o, p. Colonies on PDA from above and below after 29 days of incubation at room temperature. Scale bars: 50 μm (c, d); 20 μm (e, f); 5 μm (g–n).

#### Holotype.

GZAAS 25-0656

#### Description.

***Saprobic*** on submerged decaying wood in a freshwater stream. **Asexual morph: *Conidiomata*** on natural substratum sporodochial, scattered, gregarious, punctiform, glistening, black. ***Mycelium*** mostly superficial, partially immersed, composed of branched, smooth-walled, septate, hyaline to pale brown hyphae. ***Conidiophores*** up to 78 μm long, 3.3–4.8 μm wide, macronematous, mononematous, cylindrical, simple, septate, smooth-walled, hyaline. ***Conidiogenous cell*** 2.7–5.3 × 3.2–3.8 μm (*x̄* = 4.5 × 3.5 μm, n = 10), terminal, determinate, integrated, holoblastic, monoblastic, cylindrical, hyaline, sometimes detached with the mature conidia. ***Conidia*** 15.5–19 × 8.5–11.5 μm (*x̄* = 17 × 10.5 μm, n = 60), acrogenous, solitary, oval, obovoid to ellipsoid, aseptate, guttulate, pale brown to dark brown. **Sexual morph**: Undetermined.

#### Culture characteristics.

Conidia germinate on PDA within 18 hours, producing germ tubes from the conidial body. Colonies on PDA are circular with a flat surface and entire margin, reaching 2.5 cm in diameter after 29 days at room temperature (approximately 25 °C), and are grey or pale brown to black on both the surface and reverse sides.

#### Material examined.

China • Guizhou Province, Zunyi City, Fuxing Town, on rotting wood in a freshwater habitat, 18 April 2025, Wang-Ming Zhang, WW48 (GZAAS 25-0656, holotype), ex-type GZCC 25-0628; • Ibid., WW48.1 (GZAAS 25-0657, isotype), ex-isotype GZCC 25-0629.

#### Notes.

In the present phylogenetic analysis, *Neomonodictys subtropicus* (GZCC 25-0628 and GZCC 25-0629) formed a sister lineage to *N. muriformis* (MFLUCC 16-1136) with 96% ML and 1.00 BYPP bootstrap support (Fig. 1). Based on the base pair comparison, our isolate (GZCC 25-0628, ex-type) differs from *N. muriformis* (MFLUCC 16-1136, ex-type) by 21/593 bp for ITS (3.5%) and 10/829 bp (1.2%) for LSU. Morphologically, *Neomonodictys subtropicus* (GZAAS 25-0656) can be readily distinguished from *N. aquatica* and *N. muriformis* by its cylindrical, hyaline conidiophores, punctiform conidiomata, and aseptate conidia (Hyde et al. 2020; Huang et al. 2022). Therefore, *Neomonodictys subtropicus* is introduced here as a new species based on molecular evidence and morphological comparison.

### ﻿ Phaeoisaria guiyangensis

Taxon classificationFungiPleurothecialesPleurotheciaceae

W.M. Zhang & L. Lu sp. nov.

A6A1838C-AF51-59CA-9A95-64E296A90194

904154

Fig. 3

#### Etymology.

The specific epithet “*guiyangensis*” refers to the type locality, Guiyang City, Guizhou Province.

**Figure 3.**
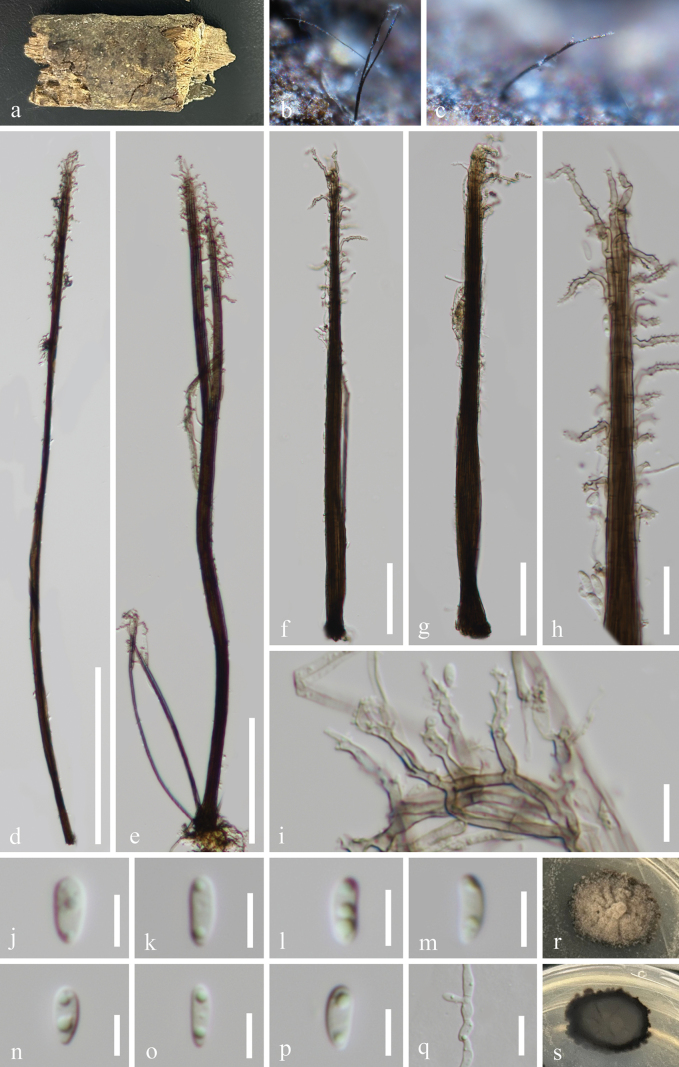
*Phaeoisaria guiyangensis* (GZAAS 25-0658, holotype). a–c. Colonies on the host surface; d–g. Synnemata; h, i. Conidiogenous cells; j–p. Conidia; q. Germinated conidium; r, s. Colonies on PDA from above and below after 35 days of incubation at room temperature. Scale bars: 200 μm (d); 100 μm (e); 50 μm (f, g); 20 μm (h); 10 μm (i, q); 5 μm (j–p).

#### Holotype.

GZAAS 25-0658

#### Description.

***Saprobic*** on submerged decaying wood in a freshwater habitat. **Asexual morph: *Colonies*** effuse, solitary, dark brown to black, hairy, covered by white conidial. ***Mycelium*** partly immersed, partly superficial, composed of septate, branched, brown hyphae. ***Synnemata*** 349–801 × 11–30.5 μm (*x̄* = 549.5 × 19.5 μm, n = 20), solitary or gathered, erect, rigid, subulate, tapering towards the apex, pale brown to black, paler towards the apex, composed of compact appressed conidiophores. ***Conidiophores*** macronematous, synnematous, septate, cylindrical, branched, straight or slightly flexuous, pale brown to black, smooth-walled. ***Conidiogenous cells*** 8–18.5 × 2–2.7 μm (*x̄* = 13.5 × 2.4 μm, n = 20), integrated, terminal and intercalary, polyblastic, fertile portion bent outwards, smooth-walled, with multiple small, hyaline, cylindrical denticulate conidiogenous loci clustered in the apical part. ***Conidia*** 6–7 × 2.1–2.8 μm (*x̄* = 6.6 × 2.4 μm, n = 30), solitary, obovoid to subcylindrical, smooth, rounded apical and obtuse basal, hyaline, aseptate, straight or slightly flexuous, guttulate. **Sexual morph**: Undetermined.

#### Culture characteristics.

Conidia germinate on PDA within 9 hours, producing germ tubes from the conidial body. Colonies on PDA are irregular with a raised surface and undulating margin, reaching 2.9 cm in diameter after 35 days at room temperature (approximately 25 °C), and are grey to dark brown on both the surface and reverse sides.

#### Material examined.

China • Guizhou Province, Guiyang City, Baiyun District, Changpo Ling National Forest Park, on rotting wood in a freshwater habitat, 15 March 2025, Wang-Ming Zhang, WW60 (GZAAS 25-0658, holotype), ex-type GZCC 25-0626; *Ibid*., WW60.1 (GZAAS 25-0659, isotype), ex-isotype GZCC 25-0627.

#### Notes.

*Phaeoisaria guiyangensis* (GZAAS 25-0658) is morphologically similar to *P. sedimenticola* (HKAS 131978) in having solitary or gathered, erect, rigid, subulate synnemata; macronematous, synnematous, septate conidiophores; terminal and intercalary, polyblastic conidiogenous cells; and solitary, obovoid to subcylindrical, hyaline, aseptate conidia (Wang et al. 2024a). However, *Phaeoisaria guiyangensis* can be distinguished from *P. sedimenticola* by its shorter conidiogenous cells (8–18.5 μm *vs.* up to 31 μm) and shorter conidia (6–7 μm *vs.* up to 10.2 μm) (Wang et al. 2024a). According to the phylogenetic analysis (Fig. 1), our new isolates formed a distinct lineage within the clade, which comprises *Phaeoisaria fasciculata* (CBS 127885 and DAOM 230055), *P. loranthacearum* (BYCDW24, BYCDW25, and CBS 140009), and *P. sparsa* (FMR 11939), indicating that GZCC 25-0626 and GZCC 25-0627 represent a distinct species. In addition, *Phaeoisaria guiyangensis* (GZAAS 25-0658) can be distinguished from *P. fasciculata* (PRM 933855) and *P. loranthacearum* (CBS 140009) by the presence of distinct synnemata, which are absent in both comparison species (Crous et al. 2015; Réblová et al. 2016). Furthermore, *Phaeoisaria guiyangensis* (GZAAS 25-0658) differs from *P. loranthacearum* (CBS 140009) and *P. sparsa* (FMR 11939) by its shorter conidia (6–7 μm *vs.* 7–8 μm and 10–15.5 μm, respectively) (Sutton 1973; Crous et al. 2015). Therefore, we propose *Phaeoisaria guiyangensis* (GZCC 25-0626 and GZCC 25-0627) as a novel species based on molecular and morphological evidence.

## ﻿Discussion

Guizhou Province is rich in fungal diversity, and annually a large number of species are introduced (Wijayawardene et al. 2021; Sun et al. 2023, 2024). However, compared to the relatively extensive research on lignicolous freshwater fungi conducted in Yunnan Province, the study of such fungi in Guizhou Province remains limited and underdeveloped. Nevertheless, as part of the ecologically diverse Yunnan–Guizhou Plateau, Guizhou harbors abundant freshwater resources and a wide range of potential fungal habitats. In recent years, increasing attention has been directed toward exploring the freshwater fungal diversity of this region, with a growing number of surveys and taxonomic studies being undertaken (Lu et al. 2018; Du et al. 2022; Liu et al. 2023; Xiao et al. 2023, 2025; Yang et al. 2023; Ma et al. 2024a, b; Shu et al. 2024). For example, studies by Liu et al. (2023) and Yang et al. (2023) have documented numerous novel species of Sordariomycetes and Dothideomycetes from lignicolous freshwater fungi collected in the characteristic karst landscapes of Guizhou Province. Moreover, helicosporous hyphomycetes are notably widespread across both freshwater and terrestrial habitats in the province, further highlighting Guizhou’s potential as an important reservoir of fungal biodiversity (Ma et al. 2024a, b; Xiao et al. 2023).

Here, we introduce two lignicolous freshwater fungal species based on comprehensive morphological and phylogenetic analyses. The discovery of these new species not only expands the known taxonomic breadth of Pleurotheciaceae but also contributes to a deeper understanding of the fungal diversity associated with freshwater ecosystems in Southwest China.

*Neomonodictys* species appear to be saprobic fungi with a preference for freshwater habitats (Hyde et al. 2020; Huang et al. 2022). To date, only two species have been reported within the genus, *N. aquatica* and *N. muriformis* (type species), both of which were isolated from freshwater habitats. Members of *Neomonodictys* are characterized by the absence of conidiophores; holoblastic, monoblastic, integrated, terminal, determinate conidiogenous cells; and acrogenous, solitary, subglobose to globose, muriform conidia (Hyde et al. 2020; Huang et al. 2022). Phylogenetic analysis of a strain isolated from submerged decaying wood in a freshwater habitat revealed a well-supported clade with the two previously described *Neomonodictys* species. However, our new collection differs morphologically from existing members of the genus in several key aspects: it possesses sporodochial, punctiform conidiomata; cylindrical, hyaline conidiogenous cells that give rise to oval, obovoid to ellipsoid, aseptate conidia, in contrast to the subglobose to globose, muriform conidia typical of *Neomonodictys* (Hyde et al. 2020; Huang et al. 2022). Based on these distinct morphological traits, the new isolate may represent a novel genus. Nonetheless, considering that *Neomonodictys* currently includes only two species, each known from a single collection, the full extent of the genus’s morphological and genetic diversity remains poorly understood. Therefore, we tentatively assign the new isolate to *Neomonodictys*, pending further studies. Future investigations, including broader sampling and molecular analyses, are warranted to clarify species boundaries within *Neomonodictys*, explore its phylogenetic placement more robustly, and uncover additional taxa that may enrich our understanding of this understudied lineage.

Given the relatively limited mycological exploration in Guizhou compared to other regions, such as Yunnan, our findings underscore the ecological significance and potential for novel taxa in this province. This study highlights the importance of continued field surveys and integrative taxonomic approaches in uncovering the hidden fungal diversity of freshwater habitats.

## Supplementary Material

XML Treatment for Neomonodictys subtropicus

XML Treatment for Phaeoisaria guiyangensis

## ﻿Additional information

### Use of AI

No use of AI was reported.

### Data availability

All of the data that support the findings of this study are available in the main text.
